# Knowledge, Attitudes, and Practices of Pulmonary Tuberculosis Patients Regarding Tuberculosis and Home-Based Treatment

**DOI:** 10.3390/healthcare14172732

**Published:** 2026-08-27

**Authors:** Xuwen Fu, Jianjie Jiang, Yuanying Li, Jiajia Duan, Zhaojing Shi, Xiang Li

**Affiliations:** 1 Department of Clinical Pharmacy, Kunming Third People’s Hospital/Yunnan Clinical Medical Center for Infectious Diseases, Kunming 650041, China; 2 Department of Internal Medicine, Kunming Third People’s Hospital/Yunnan Clinical Medical Center for Infectious Diseases, Kunming 650041, China; 3 Department of Medical Engineering, Kunming Third People’s Hospital/Yunnan Clinical Medical Center for Infectious Diseases, Kunming 650041, China; 4 Department of Radiology, Kunming Third People’s Hospital/Yunnan Clinical Medical Center for Infectious Diseases, Kunming 650041, China

**Keywords:** tuberculosis, pulmonary, health knowledge, attitudes, practice, patient compliance, self care, cross-sectional studies, surveys and questionnaires

## Abstract

**Objectives:** To assess the knowledge, attitudes, and intended practices (KAP) of pulmonary tuberculosis (PTB) patients regarding the disease and home-based treatment. **Methods:** A cross-sectional study was conducted at the authors’ hospital from 1 September 2024 to 28 February 2025, using a self-designed KAP questionnaire. Differences in KAP scores across demographic subgroups were analyzed, and the associations among knowledge, attitude, and practice were examined. **Results:** A total of 509 valid responses were included (effective rate: 90.73%). Participants were predominantly male (56.6%) and rural residents (68.0%). Their mean (SD) knowledge, attitude, and intended practice scores were 26.20 (2.90) (possible range: 15–30), 32.09 (4.14) (possible range: 9–45), and 35.89 (5.57) (possible range: 8–40), respectively. Correlation analysis showed significant but relatively weak positive correlations between knowledge and attitude (r = 0.257, *p* < 0.001), knowledge and intended practice (r = 0.136, *p* = 0.002), and attitude and intended practice (r = 0.251, *p* < 0.001). Multivariate logistic regression revealed that knowledge score (OR = 1.098, *p* = 0.007) and attitude score (OR = 1.094, *p* < 0.001) were independently associated with better intended practice. Living in rural areas (OR = 0.633, *p* = 0.043) and being government/enterprise/public institution staff (OR = 0.386, *p* = 0.004) were independently associated with lower intended practice scores. **Conclusions:** These findings highlight that higher levels of knowledge among PTB patients are positively associated with more favorable attitudes and better anticipated self-management practices. These associative findings generate the hypothesis that targeted educational interventions, especially among populations with lower education levels or non-urban backgrounds, might improve expected home-based treatment adherence, which warrants validation in future longitudinal studies.

## 1. Introduction

Pulmonary tuberculosis (PTB) remains a significant global public health challenge, ranking among the top 10 causes of death worldwide and representing the leading cause of mortality from a single infectious agent [1]. According to the World Health Organization’s recent estimates, there were approximately 10.8 million new tuberculosis cases and 1.25 million deaths globally in 2023, and China continues to rank as one of the countries with a high tuberculosis burden worldwide [2,3]. Treatment adherence is crucial for achieving cure, preventing drug resistance, and controlling disease transmission [4]. With the evolution of healthcare delivery models and the impact of events such as the COVID-19 pandemic, home-based treatment has become increasingly important in tuberculosis management, particularly after disease stabilization and during the continuation phase of treatment [5]. The success of home-based treatment fundamentally depends on patients’ self-management capabilities, treatment adherence, and appropriate infection control behaviors within the household setting [6].

The Knowledge–Attitude–Practice (KAP) theoretical framework serves as a pivotal model for understanding and influencing health behaviors, operating on the fundamental premise that knowledge positively influences attitudes, which subsequently shape individual practices. This model has been extensively utilized in healthcare domains to comprehensively assess target populations’ understanding, perceptions, and behaviors regarding specific health conditions and interventions [7]. For pulmonary tuberculosis patients, particularly those undergoing home-based treatment, adequate knowledge about their disease, positive attitudes toward treatment, and appropriate intended self-management practices are essential for successful outcomes. Given China’s substantial tuberculosis burden, investigating the KAP profile of Chinese tuberculosis patients becomes particularly crucial for developing targeted interventions and improving treatment outcomes.

Previous research has demonstrated that knowledge and beliefs, social support, and self-regulation skills significantly influence tuberculosis self-management behaviors [1]. Studies have identified tuberculosis knowledge, health education, healthcare worker support, and family support as key determinants of self-management behaviors among tuberculosis patients [8]. However, research focusing specifically on the structural KAP pathways among pulmonary tuberculosis patients receiving home-based treatment in Southwestern China remains limited. China exhibits immense geographic variations in tuberculosis management adherence, healthcare resources, and patient demographic characteristics across its different regions. Consequently, conducting systematic KAP investigations tailored to home-based patients in specific regions is crucial to provide more targeted, localized public health evidence. The structural relationships between these components, particularly the potential mediating role of attitudes in translating knowledge into intended practice, remain inadequately explored in this specific population [9,10]. Therefore, this study aims to investigate the KAP of pulmonary tuberculosis patients regarding tuberculosis and home-based treatment in Southwestern China, and analyze the associations between demographic and disease characteristics and their KAP levels. Understanding these unique pathways will provide valuable insights for developing evidence-based, region-specific educational interventions and support strategies to enhance home-based tuberculosis treatment outcomes.

## 2. Materials and Methods

### 2.1. Study Design and Participants

This cross-sectional study was conducted at Kunming Third People’s Hospital between 1 September 2024 and 28 February 2025. The day, month and year of the start of the recruitment period for this study is 1 September 2024, and the end is 28 February 2025. The study population comprised inpatients with a confirmed diagnosis of active pulmonary tuberculosis, based on the diagnostic criteria outlined in the Health Industry Standard of the People’s Republic of China: Diagnosis of Pulmonary Tuberculosis (WS 288-2017 [11]).

Participants were eligible for inclusion if they met the following criteria: (1) fully conscious and exhibiting no observable cognitive impairment; (2) capable of verbal communication and independently completing the questionnaire; and (3) willing to participate voluntarily, having provided informed consent. Exclusion criteria included: (1) diagnosis of central nervous system tuberculosis; (2) presence of comorbid psychiatric disorders; (3) severe disease requiring admission to the intensive care unit (ICU); or (4) prior participation in similar studies.

The study protocol received ethical approval from the Ethics Review Committee of Kunming Third People’s Hospital (Approval No. KSLL202408130006), and written informed consent was obtained from all participants prior to data collection. However, it should be noted that due to the cross-sectional design of this study, causal relationships between the variables cannot be inferred.

### 2.2. Research Instruments

Questionnaire design was based on relevant guidelines and literature [12,13,14,15], and its content validity was established through rigorous review and feedback from 3 experts in epidemiology and tuberculosis. A pilot study (N = 50) confirmed good overall internal consistency (Cronbach’s α = 0.833), and the specific reliability coefficients for the knowledge, attitude, and practice dimensions were 0.764, 0.764, and 0.920, respectively. To further assess construct validity and confirm the dimensional structure, Confirmatory Factor Analysis (CFA) was conducted on the full valid sample (N = 509) using the Robust Maximum Likelihood (MLR) estimator. The factor loadings for the practice dimension ranged from 0.676 to 0.848 (all *p* < 0.001). For the attitude dimension, items a2 to a6 exhibited negative loadings (−0.59 to −0.78), which appropriately reflects their reverse-coded nature. For the knowledge dimension, most loadings ranged from 0.18 to 0.70; item k7 had a lower loading (0.084, *p* = 0.084) but was purposefully retained due to its critical clinical importance regarding the standard treatment duration for drug-resistant tuberculosis. The three-factor model demonstrated a marginal but acceptable fit, with Root Mean Square Error of Approximation (RMSEA) = 0.058, Standardized Root Mean Square Residual (SRMR) = 0.079, Tucker–Lewis Index (TLI) = 0.867, and Comparative Fit Index (CFI) = 0.879 (Supplementary Figure S1, Supplementary Table S1).

The questionnaire comprised two main sections. The first section captured demographic and clinical characteristics, including age, sex, body mass index (BMI), place of residence, ethnicity, marital status, educational attainment, employment status, monthly household income, post-discharge living arrangements, diagnostic delay, and initial treatment status. In this study, diagnostic delay was defined as a period exceeding four weeks between the onset of tuberculosis-related symptoms and the patient’s first contact with a formal healthcare facility, consistent with previously published definitions [14].

The KAP scale was structured into three specific dimensions (Supplementary File S1: Final Questionnaire in English): knowledge, attitude, and practice. The knowledge dimension consisted of 15 single-choice items designed to assess patients’ understanding of basic tuberculosis and home-based treatment (e.g., routes of transmission, treatment duration, curability, details of home-based treatment). A binary scoring mechanism was applied: each correct answer scored 2 points; incorrect or “not sure” answers scored 1 point, resulting in a total score range of 15–30. The attitude dimension comprised 9 items aimed at gauging patients’ feelings and dispositions toward tuberculosis and home-based treatment (e.g., shame, confidence in cure, perceived safety of home treatment, concern for family infection, need for supervision, preference for hospitalization). These were assessed using a five-point Likert scale ranging from strongly agree to strongly disagree. Positive attitude items were scored directly from 5 (strongly agree) to 1 (strongly disagree). Conversely, negatively worded items (items 1, 7, 8, and 9) were reverse-coded (scored from 1 for strongly agree to 5 for strongly disagree) to ensure that higher aggregate scores consistently reflected more positive attitudes, yielding a total score range of 9–45. The practice dimension comprised 8 items measuring intended self-management practices and expected adherence during home-based treatment (e.g., medication adherence, not stopping treatment arbitrarily, contacting healthcare institutions, recording medication use, maintaining personal hygiene, avoiding bed sharing, handling sputum). These utilized a five-point Likert frequency scale scored directly from 5 (always) to 1 (never), with a total score range of 8–40.

### 2.3. Data Collection

Data collection was conducted using both paper-based and electronic questionnaires. The study sample comprised all hospitalized patients who met the predefined inclusion and exclusion criteria during the designated study period. Questionnaire administration was carried out by trained healthcare professionals involved in the study. Within 24 to 48 h following patient admission, a designated healthcare professional approached eligible individuals in their hospital wards and offered them a choice between completing the questionnaire in paper form or via an electronic platform. Patients were encouraged to select the format they found most convenient. During the administration process, healthcare staff provided clarification as needed to ensure that participants fully understood each item and could complete the questionnaire accurately and independently. To maintain data quality, all returned questionnaires were reviewed by research team members for completeness, internal consistency, and logical coherence of responses. The electronic version of the questionnaire was developed using the SoJump platform (SoJump.com; wjx.cn), and a QR code was generated to facilitate access. Participants opting for the electronic version scanned the code using their mobile devices to complete the survey.

### 2.4. Sample Size Calculation

The required sample size was calculated using the following formula:

n = (Z_1−α/2_/δ)^2^ × *p* × (1 − *p*)

In this equation, n represents the estimated sample size, *p* is the anticipated proportion, and δ denotes the acceptable margin of error (standard error). To obtain the maximum sample size under conditions of uncertainty, *p* was conservatively set at 0.5. The Type I error rate (α) was set at 0.05, corresponding to a Z-value of 1.96 for a 95% confidence level. The allowable margin of error (δ) was specified as 0.05. Based on these parameters, the calculated minimum sample size was adjusted for an anticipated effective response rate of 90%, yielding a final target of no fewer than 430 completed questionnaires.

### 2.5. Statistical Methods

Data analysis was performed using R software (version 4.3.2) and Stata (version 18.0). Continuous variables were summarized as means and standard deviations (mean ± SD), while categorical variables were presented as frequencies and percentages [n (%)]. To compare demographic and clinical characteristics across different KAP (Knowledge, Attitude, Practice) dimension scores, appropriate statistical tests were selected based on data distribution. For continuous variables, independent-samples *t*-tests were applied when normality assumptions were met; otherwise, the Wilcoxon–Mann–Whitney U test was used. For comparisons involving three or more groups, one-way analysis of variance (ANOVA) was conducted under the assumptions of normal distribution and homogeneity of variances, whereas the Kruskal–Wallis H test was employed for non-normally distributed data or unequal variances. Correlations among the three KAP dimensions were examined using Pearson correlation coefficients for normally distributed scores, and Spearman rank correlation coefficients when normality could not be assumed. To further assess the associations between KAP outcomes and demographic or disease-related variables, each dimension score was dichotomized at the median (i.e., above vs. at or below the median) to establish high- and low-scoring groups. This approach was explicitly selected to enhance the interpretability of the findings for public health decision-making and clinical pragmatism, thereby elevating the guiding value for targeted, precision clinical education. The dichotomized scores were subsequently treated as the dependent variables in univariate and multivariate logistic regression analyses. Variables with a *p*-value < 0.10 in univariate models were considered for inclusion in multivariate logistic regression for the knowledge and attitude dimensions, while a more inclusive threshold of *p* < 0.20 was applied for the practice dimension. Odds ratios (ORs) and their corresponding 95% confidence intervals (95% CIs) were calculated to estimate effect sizes. This study was reported in adherence to the Strengthening the Reporting of Observational Studies in Epidemiology (STROBE) guidelines for cross-sectional studies.

## 3. Results

### 3.1. Questionnaire Quality

Initially, a total of 561 participants were initially included. The following cases were excluded from the analysis: 11 participants who did not provide informed consent, 23 who completed the questionnaire in less than 120 s or more than 1800 s, and 18 who reported abnormal height or weight values. After exclusions, a final valid sample of 509 cases was included, with an effective rate of 90.73%.

### 3.2. Demographic Information on Participants

The study included 509 TB patients (56.6% male; predominantly aged 34.8% aged 18–29 years), predominantly rural residents (68.0%), Han Chinese (69.9%), and married (60.3%). Their mean (SD) knowledge, attitude, and practice scores were 26.20 (2.90), 32.09 (4.14), and 35.89 (5.57), respectively. Knowledge scores increased with higher education (*p* < 0.001), peaking among those with associate/bachelor’s degrees (27.48 ± 2.33 vs. 25.17 ± 3.09 for primary school or below). Younger patients (18–29 years) demonstrated significantly higher knowledge scores (26.63 ± 2.74 vs. 25.62 ± 2.90 in ≥50 years, *p* = 0.001). While younger patients also had higher practice scores (36.23 ± 5.64 vs. 35.75 ± 5.83), this difference was not statistically significant (*p* = 0.195). Participants with monthly household incomes ≥5000 CNY scored higher in knowledge (26.82 ± 2.63 for 5000–10,000 CNY vs. 25.86 ± 3.01 for <5000 CNY, *p* = 0.002) and attitude (32.75 ± 4.33 vs. 31.75 ± 3.98, *p* = 0.026). Employment type was significantly associated with knowledge, with government/enterprise staff scoring highest (27.42 ± 2.44 vs. 25.67 ± 2.92 for workers/farmers, *p* < 0.001). Patients living with family post-discharge reported better practice scores (36.18 ± 5.18 vs. 34.74 ± 6.82 for those living alone, *p* = 0.255). Ethnic minorities exhibited marginally higher practice scores (36.56 ± 4.66 vs. 35.60 ± 5.91, *p* = 0.607), while delayed TB diagnosis showed no significant association with KAP scores (*p* > 0.05) (Table 1). Based on patients’ previous experience (Table 1: Newly diagnosed), 65.4% (N = 333) of the patients in our sample were newly diagnosed (without prior home-based TB treatment experience), while 34.6% (N = 176) had been previously diagnosed and treated. Interestingly, our comparison showed no statistically significant differences between the newly diagnosed and previously treated subgroups across all three dimensions: knowledge (*p* = 0.710), attitude (*p* = 0.335), and intended practice (*p* = 0.590). The mean (SD) intended practice score was 35.89 (5.44) for newly diagnosed patients and 35.89 (5.84) for previously treated patients. This indicates that prior clinical or treatment experience did not significantly alter patients’ self-reported practice intentions or perceived self-efficacy.

### 3.3. Distribution of Responses to Knowledge, Attitude, and Practice

The study identified critical knowledge gaps among TB patients regarding disease transmission and home-based treatment protocols. While most recognized TB as a bacterial infection (79.17%, K1) and the necessity of continuing prescribed treatment post-discharge (85.66%, K8), significant deficits persisted. Only 44.6% knew the standard treatment duration for drug-resistant TB is 18–24 months (K7), with 29.47% uncertain, risking premature treatment cessation. Alarmingly, 35.17% incorrectly believed mask-wearing alone negates the need for >2 m distancing in shared spaces (K13), and 61.49% opposed bed-sharing with family members before sputum negativity (K12), indicating poor transmission prevention awareness. Furthermore, 21.22% underestimated the standard treatment duration for regular TB (6–8 months, K6), and 18.07% endorsed self-adjusting medication dosages (K10), posing risks for drug resistance. Despite high awareness of TB’s curability (86.05%, K4), 17.29% remained unsure about isolation practices (K12) (Supplementary Table S2).

Responses to the attitude dimension showed that 23.77% strongly agreed and 31.04% agreed that they prefer to complete treatment in a hospital rather than at home if conditions allow (A9), 22.2% strongly agreed and 18.07% agreed that they find it difficult to strictly follow the treatment plan during home-based treatment without close supervision by a doctor (A7), and 19.65% strongly agreed and 26.52% agreed that home-based treatment may lack proper infection control and pose a risk of transmitting the disease to their family (A8) (Supplementary Table S3).

Responses to the intended practice dimension showed that, with the exception of taking medication as required by their doctor (P1) and paying attention to personal hygiene (P6), no more than 70% of the participants were able to firmly intend to practice the other items; specifically, only 62.08% always kept a covered sputum cup or spittoon with disinfectant at home (P8), and only 64.44% always took their medication without forgetting (P2) (Supplementary Table S4).

### 3.4. Correlations Between KAP

Correlation analysis showed that there were significant but relatively weak positive correlations between knowledge and attitude (r = 0.257, *p* < 0.001) as well as between knowledge and practice (r = 0.136, *p* = 0.002). Also, there was a significant but relatively weak positive correlation between attitude and practice (r = 0.251, *p* < 0.001) (Table 2).

### 3.5. Factors Associated with KAP

Multivariate logistic regression showed that with BMI of 18.50–23.99 kg/m^2^ (OR = 0.442, 95% CI: [0.269, 0.726], *p* = 0.001), with BMI of ≥28.00 kg/m^2^ (OR = 0.395, 95% CI: [0.168, 0.931], *p* = 0.034), and with associate degree/bachelor’s degree (OR = 4.960, 95% CI: [2.321, 10.599], *p* < 0.001) were independently associated with knowledge (Table 3). Concurrently, knowledge score (OR = 1.171, 95% CI: [1.091, 1.256], *p* < 0.001) was independently associated with positive attitude (Table 4). Moreover, knowledge score (OR = 1.098, 95% CI: [1.026, 1.175], *p* = 0.007), attitude score (OR = 1.094, 95% CI: [1.043, 1.148], *p* < 0.001), living in rural areas (OR = 0.633, 95% CI: [0.407, 0.986], *p* = 0.043), and being government, enterprise, or public institution staff (OR = 0.386, 95% CI: [0.201, 0.743], *p* = 0.004) were independently associated with intended practice (Table 5).

## 4. Discussion

PTB patients demonstrated generally adequate knowledge, positive attitudes, and proactive intended practices toward the disease and its home-based management, with higher education levels and normal BMI significantly associated with better knowledge, and both knowledge and attitude independently associated with improved intended practices. We hypothesize that interventions aiming to strengthen patient education, particularly among individuals with lower education or from rural areas, might improve anticipated adherence to home-based TB treatment, a premise that requires future interventional research.

The analysis revealed a consistent pattern: patients with greater knowledge about tuberculosis were more likely to exhibit positive attitudes and report intended treatment-supportive behaviors. Although the correlations among these variables were relatively weak, attitude was a statistically significant predictor of behavior, and its role appears to be interrelated with the patients’ knowledge base. This multidimensional linkage is broadly compatible with conceptual frameworks in patient education and behavioral health, where informed patients tend to engage more deliberately with chronic disease management [15,16]. The findings suggest that targeting knowledge deficits may serve as a foundational step in strengthening both attitudes and behavioral compliance, particularly in self-managed care contexts.

Further support for these dynamics emerged from the multivariate regression analyses. Even after adjusting for sociodemographic variables, knowledge remained significantly associated with both attitude and intended practice, and attitude continued to show an independent association with intended practice. These associations align with earlier findings in chronic disease contexts, where health education was linked to more favorable behaviors and attitudinal shifts [17,18]. In this study, however, the strength of the knowledge-intended practice pathway was comparatively attenuated, raising the possibility that knowledge alone may be insufficient unless supported by confidence, motivation, and perceived relevance. This distinction holds practical relevance in designing intervention strategies that address both the cognitive and affective dimensions of patient behavior.

The role of educational attainment was especially prominent. Individuals with higher education levels were more likely to score well on the knowledge scale, a result that is consistent with previous work linking formal education to health literacy. This relationship retained significance even after controlling for other factors, suggesting a potentially independent association. The practical implication is not limited to formal schooling alone but rather points to the cumulative advantage of exposure to structured learning environments, which may foster not only information retention but also greater trust in medical recommendations [19,20].

Intended behavioral outcomes were also associated with structural conditions. Participants from rural areas were less likely to report intentions to engage in recommended practices, despite comparable knowledge and attitude scores in the unadjusted analysis. This disparity, clarified in multivariate models, may suggest latent access or system trust barriers not captured through self-reported cognitive metrics. Patients working in public institutions also showed a lower likelihood of high intended practice scores, a finding that diverges from expectations based on occupational access to health information. While in similar settings, healthcare adherence among government employees has often been attributed to institutional exposure and routine-based lifestyles [21,22], our current finding suggests potential contextual nuances. We hypothesize this might reflect variations in work-related stress, schedule flexibility, or reliance on institutional care over self-management. However, these explanations remain speculative. Because our study did not include variables measuring occupational workload, time flexibility, workplace cultural characteristics, or healthcare accessibility, this aspect of the discussion should be interpreted cautiously and warrants further focused investigation.

Closer examination of item-level responses offered further insight. While general awareness of TB transmission and treatment was high, misconceptions were concentrated around more complex or ambiguous topics, such as the appropriate response to symptom fluctuation and environmental infection control measures at home. These response patterns suggest that while patients may grasp the basics of TB, there remains confusion about specific management scenarios. Knowledge items involving treatment duration, mask use, and the necessity of distancing measures in shared spaces were particularly prone to error. Similar gaps have been reported in other contexts where discharge education is inconsistently delivered or overly generalized [23,24].

Attitudinal responses were more variable. Most participants expressed optimism regarding treatment efficacy and a willingness to adhere to home-based regimens. Nonetheless, a substantial proportion remained ambivalent, particularly regarding the perceived safety and adequacy of home care. Expressions of discomfort with unsupervised treatment and concern about infection control were not uncommon. These attitudes may reflect residual doubts rooted in cultural expectations about institutional care or in previous experiences with health services. In studies conducted in Asian regions, preference for hospital-based treatment has been associated with stronger perceptions of medical authority and risk containment [25,26].

While reported practices were largely positive, certain behaviors involving coordination with external institutions—such as contacting health authorities or recording medication use—were less consistently observed. This discrepancy may be linked to logistical challenges or limited trust in community-level health infrastructure. The relatively high adherence to individual hygiene practices suggests that patients are capable of integrating recommendations into daily life, provided those behaviors are straightforward and personally manageable. However, system-facing tasks may require greater support and reassurance. This divergence has been noted in other decentralized care settings, where the burden of self-monitoring and reporting can erode engagement unless supported by community-based follow-up or digital aids [27,28].

Drawing from these associations, we propose several hypotheses for future interventional studies. First, whether tailoring patient education efforts to address identified conceptual gaps, rather than relying on general information delivery, yields better outcomes needs to be tested. Instruction could potentially be more effective if it clarifies specific points of confusion, such as when to seek medical advice, how to adjust home routines to reduce infection risk, and the rationale behind fixed treatment durations. Second, future research should explore whether education strategies accounting for educational and geographic disparities, such as using visual tools, simplified messaging, and peer-led models for individuals with lower formal education or from rural areas, are more effective than written instructions alone. Third, the potential benefit of enhancing continuity between hospital discharge and home-based treatment, through standardized follow-up, mobile health prompts, and consistent contact with community health workers to bridge implementation gaps, should be evaluated in prospective trials [29,30].

This study has several limitations. First, as a cross-sectional design was used, causal relationships between knowledge, attitudes, and practices could not be established. Second, the data were collected from a single hospital in Kunming, which may limit the generalizability of the findings to broader populations. Third, the use of self-reported questionnaires may have introduced social desirability bias. Given that the survey was conducted in a hospital setting and administered by healthcare professionals, patients might have tended to overreport their compliance behaviors to align with medical norms. Furthermore, because both groups of patients were surveyed in hospital wards within 24 to 48 h of admission, the responses to the “practice” dimension primarily measure intended self-management practices, expected adherence, or perceived self-efficacy in home-based treatment, rather than directly observed, actual, and ongoing home adherence. However, as our subgroup analysis indicated, there were no significant differences in intended practice scores between newly diagnosed and previously treated patients, suggesting that prior experience did not substantially alter these anticipated behaviors. This experiential and spatial disconnect implies that the self-reported data may not fully capture instances of missed doses or non-compliant behaviors in the complex, real-world home setting once patients are completely free from direct medical supervision. Finally, our statistical and scoring methodologies entail certain limitations that must be acknowledged. The knowledge scoring system assigned 1 point to incorrect or not sure responses, creating a floor effect (minimum score of 15 instead of 0) that may artificially overestimate patients’ absolute knowledge levels. However, because we utilized median dichotomization for our regression analyses, this uniform shift does not alter the relative ranking of the participants. Additionally, while dichotomizing continuous KAP scores at the median for logistic regression created nearly 1:1 balanced groups to maximize the statistical power of the models and provide robust parameter estimates, we explicitly acknowledge that this approach inherently entails a loss of some statistical information compared to analyzing the data as continuous variables.

In summary, pulmonary tuberculosis patients demonstrated generally sufficient knowledge, favorable attitudes, and relatively sound intended self-management practices, with clear associative relationships observed among these dimensions. Given these findings, we hypothesize that targeted educational interventions, particularly among individuals with lower educational levels, urban employment backgrounds, or residing in rural areas, might foster better patient engagement in home-based care; however, this remains to be confirmed by future prospective studies.

## 5. Conclusions

In conclusion, this cross-sectional study demonstrates that among pulmonary tuberculosis patients, higher levels of disease-specific knowledge are positively associated with more favorable attitudes and better anticipated home-based management practices. Attitude also serves as an independent correlate of intended practice. These associative findings lead to the hypothesis that targeted educational interventions, especially those designed for populations with lower educational attainment or non-urban backgrounds, might enhance expected home-based treatment adherence, which must be tested in future interventional studies. However, given the cross-sectional nature of this research, causal inferences cannot be drawn, and future longitudinal studies are warranted to further investigate these relationships.

## Figures and Tables

**Table 1:** Demographic characteristics and KAP scores.

N = 509	N (%)	Knowledge	*p*	Attitude	*p*	Practice	*p*
		Mean (SD)		Mean (SD)		Mean (SD)	
Total score	509 (100.0)	26.20 (2.90)		32.09 (4.14)		35.89 (5.57)	
Age			0.001		0.279		0.195
18–29	177 (34.8)	26.63 (2.74)		32.24 (4.32)		36.23 (5.64)	
30–49	185 (36.3)	26.25 (3.00)		32.34 (4.37)		35.68 (5.32)	
50 and more	147 (28.9)	25.62 (2.90)		31.62 (3.56)		35.75 (5.83)	
BMI			0.104		0.345		0.538
<18.5	114 (22.4)	26.48 (3.07)		32.46 (4.34)		36.37 (5.63)	
18.50–23.99	250 (49.1)	25.98 (2.92)		31.94 (4.17)		35.85 (5.31)	
24.00–27.99	114 (22.4)	26.49 (2.71)		32.26 (3.91)		35.65 (5.99)	
≥28.00	31 (6.1)	25.87 (2.80)		31.35 (3.98)		35.32 (6.01)	
Gender			0.254		0.154		0.497
Male	288 (56.6)	26.08 (2.93)		31.85 (3.98)		35.70 (5.80)	
Female	221 (43.4)	26.37 (2.87)		32.42 (4.32)		36.13 (5.26)	
Residence			0.210		0.412		0.352
Urban	163 (32.0)	26.58 (2.40)		32.49 (4.85)		36.17 (5.53)	
Rural	346 (68.0)	26.03 (3.10)		31.91 (3.75)		35.76 (5.60)	
Ethnicity			0.219		0.061		0.607
Han	356 (69.9)	26.09 (2.97)		31.93 (4.23)		35.60 (5.91)	
Ethnic minority	153 (30.1)	26.46 (2.74)		32.48 (3.90)		36.56 (4.66)	
Marital status			0.084		0.408		0.197
Single	169 (33.2)	26.46 (2.95)		32.58 (4.36)		36.43 (5.43)	
Married	307 (60.3)	26.12 (2.86)		31.83 (4.01)		35.58 (5.66)	
Divorced or separated	22 (4.3)	26.00 (3.34)		31.95 (4.54)		36.05 (6.00)	
Widowed	11 (2.2)	25.00 (2.28)		32.27 (3.00)		36.00 (4.40)	
Education			<0.001		0.011		0.622
Primary school or below	135 (26.5)	25.17 (3.09)		31.58 (3.95)		35.91 (5.69)	
Junior high school	131 (25.7)	25.79 (2.87)		31.62 (3.80)		35.25 (6.19)	
Senior high school/Vocational school	93 (18.3)	26.22 (2.77)		32.11 (4.28)		36.17 (5.88)	
Associate degree/Bachelor’s degree	150 (29.5)	27.48 (2.33)		32.97 (4.38)		36.25 (4.63)	
Current employment			<0.001		0.063		0.756
Worker/Farmer	236 (46.4)	25.67 (2.92)		31.70 (3.64)		35.77 (5.81)	
Self-employed	35 (6.9)	26.46 (2.47)		33.63 (4.42)		37.00 (3.68)	
Government, enterprise, or public institution staff	66 (13.0)	27.42 (2.44)		32.68 (4.45)		35.68 (4.92)	
Student	60 (11.8)	26.60 (3.11)		31.52 (3.83)		35.32 (6.50)	
Other	112 (22.0)	26.32 (2.89)		32.41 (4.82)		36.22 (5.42)	
Monthly household income			0.002		0.026		0.708
<5000	341 (67.0)	25.86 (3.01)		31.75 (3.98)		35.90 (5.58)	
5000–10,000	138 (27.1)	26.82 (2.63)		32.75 (4.33)		35.88 (5.77)	
10,001–20,000	22 (4.3)	27.36 (2.11)		32.91 (4.56)		36.41 (4.38)	
>20,000	8 (1.6)	26.88 (2.10)		33.12 (5.03)		34.38 (5.63)	
Living situation after discharge			0.747		0.761		0.255
Living alone	103 (20.2)	25.93 (3.36)		32.04 (4.25)		34.74 (6.82)	
Living with family	406 (79.8)	26.27 (2.78)		32.11 (4.11)		36.18 (5.18)	
Delay in tuberculosis diagnosis			0.635		0.904		0.156
No	336 (66.0)	26.19 (3.00)		32.12 (4.21)		35.65 (5.72)	
Yes	173 (34.0)	26.22 (2.71)		32.03 (4.01)		36.36 (5.27)	
Newly diagnosed			0.710		0.335		0.590
Yes	333 (65.4)	26.20 (2.99)		32.23 (4.08)		35.89 (5.44)	
No	176 (34.6)	26.21 (2.74)		31.84 (4.25)		35.89 (5.84)	

**Table 2:** Correlation analysis.

Spearman	Knowledge	Attitude	Practice
Knowledge	1.000		
Attitude	0.257 (*p* < 0.001)	1.000	
Practice	0.136 (*p* = 0.002)	0.251 (*p* < 0.001)	1.000

**Table 3:** Univariate and multivariate logistic regression for knowledge.

Knowledge Ksum ≥ 27,272 (53.44%) Ksum ≤ 26,237 (46.56%)	Univariate Analysis	*p*	Multivariate Analysis	*p*
	OR (95%CI)		OR (95%CI)	
Age				
18–29				
30–49	0.878 (0.577, 1.335)	0.542	1.172 (0.627, 2.192)	0.620
50 and more	0.439 (0.280, 0.684)	<0.001	0.831 (0.400, 1.724)	0.618
BMI				
<18.5				
18.50–23.99	0.527 (0.332, 0.828)	0.006	0.442 (0.269, 0.726)	0.001
24.00–27.99	0.719 (0.421, 1.222)	0.224	0.589 (0.326, 1.064)	0.080
≥28.00	0.463 (0.204, 1.031)	0.060	0.395 (0.168, 0.931)	0.034
Gender				
Male				
Female	1.063 (0.748, 1.512)	0.733		
Residence				
Urban				
Rural	0.868 (0.596, 1.261)	0.458		
Ethnicity				
Han				
Ethnic minority	1.315 (0.898, 1.932)	0.161		
Marital status				
Single				
Married	0.705 (0.481, 1.029)	0.071	1.081 (0.586, 1.996)	0.803
Divorced or separated	0.808 (0.330, 2.014)	0.640	1.296 (0.455, 3.692)	0.627
Widowed	0.150 (0.022, 0.603)	0.017	0.331 (0.060, 1.836)	0.206
Education				
Primary school or below				
Junior high school	1.731 (1.061, 2.840)	0.029	1.559 (0.917, 2.650)	0.101
Senior high school/Vocational school	2.299 (1.344, 3.966)	0.003	1.983 (1.000, 3.934)	0.050
Associate degree/Bachelor’s degree	4.661 (2.843, 7.758)	<0.001	4.960 (2.321, 10.599)	<0.001
Current employment				
Worker/Farmer				
Self-employed	2.475 (1.196, 5.362)	0.017	1.450 (0.621, 3.386)	0.391
Government, enterprise, or public institution staff	2.767 (1.569, 5.012)	0.001	0.845 (0.386, 1.846)	0.672
Student	1.937 (1.094, 3.483)	0.025	0.681 (0.293, 1.582)	0.372
Other	1.786 (1.136, 2.825)	0.013	1.001 (0.569, 1.761)	0.997
Monthly household income				
<5000				
5000–10,000	2.024 (1.349, 3.065)	0.001	1.115 (0.674, 1.844)	0.671
10,001–20,000	1.889 (0.788, 4.837)	0.163	0.860 (0.318, 2.326)	0.767
>20,000	1.079 (0.252, 4.631)	0.915	0.636 (0.139, 2.924)	0.561
Living situation after discharge				
Living alone				
Living with family	0.954 (0.617, 1.471)	0.832		
Delay in tuberculosis diagnosis				
No				
Yes	0.886 (0.613, 1.280)	0.518		
Newly diagnosed				
Yes				
No	0.931 (0.646, 1.343)	0.702		

Note: The modeled outcome is the dichotomized knowledge score (above median vs. at or below median). The reference category for each categorical variable is the first listed subgroup. Variables were selected for inclusion in the multivariate model if their *p*-value was <0.10 in the univariate analysis. Missing data were not present, as all 509 included responses were complete and valid.

**Table 4:** Univariate and multivariate logistic regression for attitude.

Attitude Asum ≥ 32,259 (50.88%) Asum ≤ 31,250 (49.12%)	Univariate Analysis	*p*	Multivariate Analysis	*p*
	OR (95%CI)		**OR (95%CI)**	
Knowledge	1.194 (1.117, 1.276)	<0.001	1.171 (1.091, 1.256)	<0.001
Age				
18–29				
30–49	0.974 (0.645, 1.472)	0.901		
50 and more	0.821 (0.529, 1.272)	0.378		
BMI				
<18.5				
18.50–23.99	0.762 (0.488, 1.187)	0.230		
24.00–27.99	1.074 (0.637, 1.812)	0.790		
≥28.00	0.691 (0.307, 1.531)	0.363		
Gender				
Male				
Female	1.233 (0.868, 1.754)	0.242		
Residence				
Urban				
Rural	0.895 (0.616, 1.300)	0.561		
Ethnicity				
Han				
Ethnic minority	1.465 (1.001, 2.151)	0.050	1.405 (0.939, 2.102)	0.098
Marital status				
Single				
Married	0.939 (0.644, 1.367)	0.741		
Divorced or separated	0.921 (0.375, 2.261)	0.855		
Widowed	0.767 (0.214, 2.640)	0.671		
Education				
Primary school or below				
Junior high school	1.056 (0.651, 1.714)	0.824	0.950 (0.567, 1.593)	0.846
Senior high school/Vocational school	1.277 (0.752, 2.173)	0.365	1.090 (0.564, 2.107)	0.797
Associate degree/Bachelor’s degree	1.983 (1.239, 3.192)	0.005	1.472 (0.724, 2.993)	0.285
Current employment				
Worker/Farmer				
Self-employed	2.122 (1.026, 4.595)	0.047	1.487 (0.642, 3.442)	0.354
Government, enterprise, or public institution staff	1.703 (0.982, 2.996)	0.060	0.746 (0.350, 1.591)	0.448
Student	0.906 (0.510, 1.598)	0.734	0.511 (0.247, 1.057)	0.070
Other	1.147 (0.732, 1.802)	0.549	0.828 (0.483, 1.418)	0.491
Monthly household income				
<5000				
5000–10,000	1.769 (1.186, 2.653)	0.005	1.423 (0.875, 2.316)	0.155
10,001–20,000	2.511 (1.032, 6.720)	0.050	1.897 (0.698, 5.157)	0.209
>20,000	1.172 (0.273, 5.029)	0.825	0.970 (0.228, 4.117)	0.967
Living situation after discharge				
Living alone				
Living with family	1.071 (0.695, 1.653)	0.756		
Delay in tuberculosis diagnosis				
No				
Yes	0.810 (0.560, 1.168)	0.259		
Newly diagnosed				
Yes				
No	0.915 (0.635, 1.319)	0.634		

Note: The modeled outcome is the dichotomized knowledge score (above median vs. at or below median). The reference category for each categorical variable is the first listed subgroup. Variables were selected for inclusion in the multivariate model if their *p*-value was <0.10 in the univariate analysis. Missing data were not present, as all 509 included responses were complete and valid.

**Table 5:** Univariate and multivariate logistic regression for practice.

Practice Psum ≥ 39,263 (51.67%) Psum ≤ 38,246 (48.33%)	Univariate Analysis	*p*	Multivariate Analysis	*p*
	OR (95%CI)		OR (95%CI)	
Knowledge	1.114 (1.047, 1.186)	0.007	1.098 (1.026, 1.175)	0.007
Attitude	1.106 (1.057, 1.157)	<0.001	1.094 (1.043, 1.148)	<0.001
Age				
18–29				
30–49	0.730 (0.482, 1.104)	0.137	0.777 (0.426, 1.418)	0.411
50 and more	0.821 (0.529, 1.273)	0.378	0.973 (0.486, 1.951)	0.939
BMI				
<18.5				
18.50–23.99	0.693 (0.442, 1.082)	0.108	0.726 (0.450, 1.171)	0.189
24.00–27.99	0.780 (0.462, 1.315)	0.352	0.807 (0.457, 1.426)	0.461
≥28.00	0.776 (0.348, 1.732)	0.532	0.987 (0.423, 2.304)	0.975
Gender				
Male				
Female	1.130 (0.796, 1.606)	0.496		
Residence				
Urban				
Rural	0.782 (0.537, 1.136)	0.198	0.633 (0.407, 0.986)	0.043
Ethnicity				
Han				
Ethnic minority	0.926 (0.634, 1.353)	0.691		
Marital status				
Single				
Married	0.744 (0.509, 1.084)	0.125	0.759 (0.417, 1.383)	0.368
Divorced or separated	1.125 (0.460, 2.861)	0.798	1.107 (0.390, 3.146)	0.848
Widowed	0.649 (0.181, 2.235)	0.489	0.530 (0.130, 2.154)	0.375
Education				
Primary school or below				
Junior high school	0.741 (0.456, 1.199)	0.222		
Senior high school/Vocational school	1.046 (0.615, 1.783)	0.870		
Associate degree/Bachelor’s degree	0.824 (0.516, 1.313)	0.417		
Current employment				
Worker/Farmer				
Self-employed	0.973 (0.477, 1.993)	0.939	0.637 (0.290, 1.396)	0.260
Government, enterprise, or public institution staff	0.677 (0.388, 1.171)	0.165	0.386 (0.201, 0.743)	0.004
Student	0.919 (0.520, 1.622)	0.769	0.552 (0.256, 1.189)	0.129
Other	1.225 (0.780, 1.933)	0.380	0.855 (0.497, 1.473)	0.573
Monthly household income				
<5000				
5000–10,000	0.932 (0.627, 1.385)	0.726		
10,001–20,000	0.905 (0.378, 2.168)	0.821		
>20,000	0.543 (0.110, 2.248)	0.408		
Living situation after discharge				
Living alone				
Living with family	1.170 (0.759, 1.806)	0.477		
Delay in tuberculosis diagnosis				
No				
Yes	1.218 (0.844, 1.762)	0.294		
Newly diagnosed				
Yes				
No	1.235 (0.857, 1.785)	0.259		

Note: The modeled outcome is the dichotomized knowledge score (above median vs. at or below median). The reference category for each categorical variable is the first listed subgroup. Variables were selected for inclusion in the multivariate model if their *p*-value was <0.10 in the univariate analysis. Missing data were not present, as all 509 included responses were complete and valid.

## Data Availability

The data presented in this study are available upon request from the corresponding author. The data are not publicly available due to privacy restrictions.

## Supplementary Materials

The following supporting information can be downloaded at https://www.mdpi.com/article/10.3390/healthcare14172732/s1. Supplementary Figure S1: Path diagram of Confirmatory Factor Analysis. Supplementary Table S1: Fit indices and pathway coefficients of Confirmatory Factor Analysis. Supplementary Table S2: Distribution of knowledge dimension responses. Supplementary Table S3: Distribution of attitude dimension responses. Supplementary Table S4: Distribution of practice dimension responses. Supplementary File S1: Final Questionnaire in English.
