# Supplementary material for: Knowledge, Attitudes, and Practices of Pulmonary Tuberculosis Patients Regarding Tuberculosis and Home-Based Treatment
Source: Healthcare (Basel). 2026 Aug 27;14(17):2732. doi: 10.3390/healthcare14172732 (PMC13565246; doi:10.3390/healthcare14172732)
Supplement: Supplementary file 1 [file healthcare-14-02732-s001.zip › healthcare-4413577 - Supplementary Figure and Tables-edited.pdf]

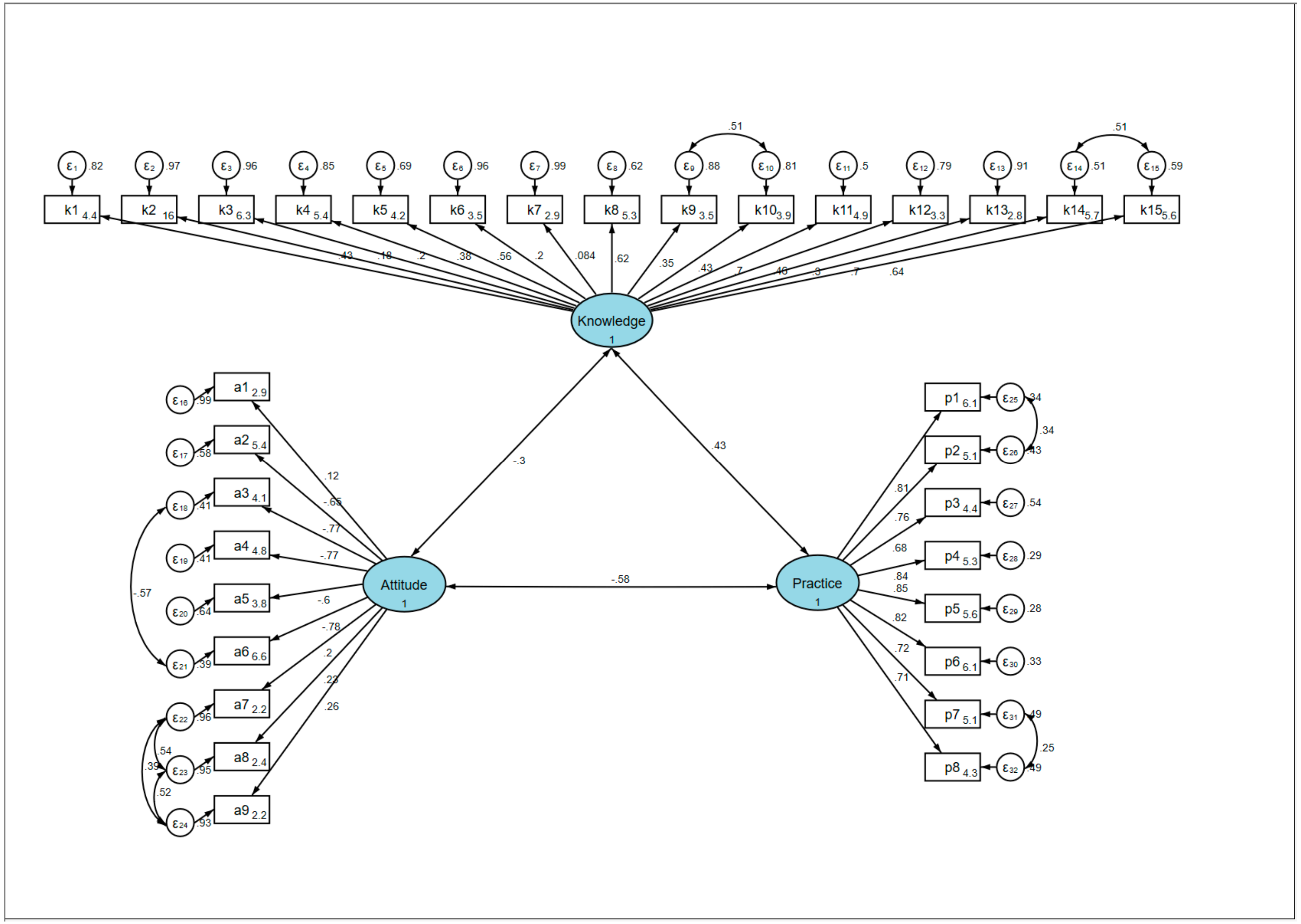

Supplementary Figure S1. Path diagram of Confirmatory Factor Analysis

Supplementary Table S1. Fit indices and pathway coefficients of Confirmatory Factor Analysis

Indicators	Reference	Results
RMSEA	<0.080	0.058
SRMR	<0.080	0.079
TLI	>0.800	0.867
CFI	>0.800	0.879

Pathway		Coefficient	std. err.	z	P>z	[95% conf. interval]	
k1	Knowledge	0.425583	0.041446	10.27	<0.001	0.344351	0.506816
k2	Knowledge	0.181905	0.047733	3.81	<0.001	0.088351	0.275459
k3	Knowledge	0.203594	0.047386	4.3	<0.001	0.11072	0.296469
k4	Knowledge	0.382476	0.043047	8.89	<0.001	0.298106	0.466846
k5	Knowledge	0.560531	0.03598	15.58	<0.001	0.490011	0.631051
k6	Knowledge	0.20477	0.047292	4.33	<0.001	0.112079	0.297462
k7	Knowledge	0.084481	0.048835	1.73	0.084	-0.01123	0.180196
k8	Knowledge	0.619079	0.033172	18.66	<0.001	0.554063	0.684096
k9	Knowledge	0.351952	0.04443	7.92	<0.001	0.26487	0.439034
k10	Knowledge	0.430406	0.04183	10.29	<0.001	0.348421	0.512392
k11	Knowledge	0.704351	0.028899	24.37	<0.001	0.64771	0.760992
k12	Knowledge	0.4597	0.040667	11.3	<0.001	0.379994	0.539407
k13	Knowledge	0.301679	0.045734	6.6	<0.001	0.212042	0.391315
k14	Knowledge	0.699224	0.029536	23.67	<0.001	0.641335	0.757113
k15	Knowledge	0.638435	0.032959	19.37	<0.001	0.573837	0.703034
a1	Attitude	0.117868	0.046042	2.56	0.01	0.027627	0.208108
a2	Attitude	-0.64668	0.028559	-22.64	<0.001	-0.70266	-0.59071
a3	Attitude	-0.76635	0.026012	-29.46	<0.001	-0.81734	-0.71537
a4	Attitude	-0.76665	0.023048	-33.26	<0.001	-0.81182	-0.72147
a5	Attitude	-0.59587	0.031599	-18.86	<0.001	-0.65781	-0.53394

a6	Attitude	-0.78388	0.025997	-30.15	<0.001	-0.83483	-0.73293
a7	Attitude	0.199772	0.044907	4.45	<0.001	0.111756	0.287787
a8	Attitude	0.230259	0.044136	5.22	<0.001	0.143753	0.316765
a9	Attitude	0.255849	0.043669	5.86	<0.001	0.17026	0.341438
p1	Practice	0.813934	0.01731	47.02	<0.001	0.780007	0.847862
p2	Practice	0.757534	0.02121	35.72	<0.001	0.715963	0.799104
p3	Practice	0.676037	0.025994	26.01	<0.001	0.625089	0.726985
p4	Practice	0.840652	0.015391	54.62	<0.001	0.810486	0.870817
p5	Practice	0.847781	0.014864	57.04	<0.001	0.818648	0.876914
p6	Practice	0.818482	0.016939	48.32	<0.001	0.785281	0.851682
p7	Practice	0.717493	0.023758	30.2	<0.001	0.670929	0.764058
p8	Practice	0.712726	0.023915	29.8	<0.001	0.665853	0.7596

Supplementary Table S2. Distribution of knowledge dimension responses

**Knowledge**	Option a	Option b	Option c
1. Tuberculosis is an infectious disease caused by bacteria.	403 (79.17%)	14 (2.75%)	92 (18.07%)
2. Which part of the body is most commonly affected by tuberculosis?	7 (1.38%)	501 (98.43%)	1 (0.2%)
3. What is the main route of transmission for pulmonary tuberculosis?	28 (5.5%)	458 (89.98%)	23 (4.52%)
4. Can pulmonary tuberculosis be cured?	438 (86.05%)	11 (2.16%)	60 (11.79%)
5. Can a person with smear-positive pulmonary tuberculosis transmit the disease to friends or family members?	394 (77.41%)	23 (4.52%)	92 (18.07%)
6. How long is the standard treatment period for regular pulmonary tuberculosis?	108 (21.22%)	332 (65.23%)	69 (13.56%)
7. How long is the standard treatment period for drug-resistant tuberculosis?	132 (25.93%)	227 (44.6%)	150(9.47%)
8. Tuberculosis patients who are discharged from the hospital before completing their treatment course should continue the anti-tuberculosis treatment plan prescribed during hospitalization at home.	436 (85.66%)	14 (2.75%)	59 (11.59%)
9. During home-based treatment, patients do not need medical guidance from healthcare professionals.	109 (21.41%)	339 (66.6%)	61 (11.98%)
10. During home-based treatment, patients can adjust their medication dosage by	92 (18.07%)	369 (72.5%)	48 (9.43%)

themselves if symptoms improve or worsen.			
11. If a patient’s home-based treatment is not properly managed, it may lead to treatment failure or the development of drug-resistant tuberculosis.	424 (83.3%)	9 (1.77%)	76 (14.93%)
12. Can an infectious tuberculosis patient share a bed with family members before their sputum tests turn negative?	108 (21.22%)	313 (61.49%)	88 (17.29%)
13. During home-based treatment, if an active tuberculosis patient wears a mask in shared household areas, maintaining a distance of more than 2 meters is unnecessary.	244 (47.94%)	179 (35.17%)	86 (16.9%)
14. Patients should take measures during home-based treatment to prevent and reduce the transmission of tuberculosis to close household contacts.	446 (87.62%)	7 (1.38%)	56 (11%)
15. Timely disinfection of the living environment and waste during home-based treatment can reduce the risk of transmission to family members and the community.	443 (87.03%)	9 (1.77%)	57 (11.2%)

Supplementary Table S3. Distribution of attitude dimension responses

**Attitude**	Strongly agree	Agree	Neutral	Disagree	Strongly disagree
1. I feel ashamed because I have tuberculosis.	60 (11.79%)	37 (7.27%)	101 (19.84%)	222 (43.61%)	89 (17.49%)
2. I believe my tuberculosis can be completely cured.	258 (50.69%)	191 (37.52%)	47 (9.23%)	6 (1.18%)	7 (1.38%)
3. I feel confident in the safety of home-based treatment and am not worried about my condition worsening.	175 (34.38%)	176 (34.58%)	118 (23.18%)	34 (6.68%)	6 (1.18%)
4. I believe that as long as I strictly follow the treatment plan prescribed by my doctor, home-based treatment can ensure timely recovery.	200 (39.29%)	204 (40.08%)	86 (16.9%)	15 (2.95%)	4 (0.79%)
5. I believe that home-based treatment can help reduce the burden on healthcare resources to some extent.	156 (30.65%)	188 (36.94%)	104 (20.43%)	53 (10.41%)	8 (1.57%)
6. I am willing to actively follow the doctor's recommendations, take my medications on time, and attend regular follow-up visits.	289 (56.78%)	186 (36.54%)	28 (5.5%)	3 (0.59%)	3 (0.59%)

7. Without close supervision by a doctor, I find it difficult to strictly follow the treatment plan during home-based treatment.	113 (22.2%)	92 (18.07%)	92 (18.07%)	167 (32.81%)	45 (8.84%)
8. I believe that home-based treatment may lack proper infection control and pose a risk of transmitting the disease to my family.	100 (19.65%)	135 (26.52%)	174 (34.18%)	81 (15.91%)	19 (3.73%)
9. If conditions allow, I would prefer to complete treatment in a hospital rather than at home.	121 (23.77%)	158 (31.04%)	153 (30.06%)	61 (11.98%)	16 (3.14%)

Supplementary Table S4. Distribution of practice dimension responses

Practice	Always able to do	Usually able to do	Not sure	Sometimes unable to do	Completely unable to do
1. During home-based treatment, I take my medication exactly as prescribed by my doctor.	376 (73.87%)	84 (16.5%)	35 (6.88%)	12 (2.36%)	2 (0.39%)
2. During home-based treatment, I never forget to take my medication.	328 (64.44%)	105 (20.63%)	52 (10.22%)	21 (4.13%)	3 (0.59%)
3. During home-based treatment, I do not stop taking medication just because my condition improves or worsens.	338 (66.4%)	84 (16.5%)	55 (10.81%)	18 (3.54%)	14 (2.75%)
4. During home-based treatment, I will promptly contact the appropriate local institution if a treatment change is needed.	343 (67.39%)	98 (19.25%)	51 (10.02%)	11 (2.16%)	6 (1.18%)
5. During home-based treatment, I cooperate with relevant personnel to document my medication use in a timely manner.	350 (68.76%)	97 (19.06%)	47 (9.23%)	12 (2.36%)	3 (0.59%)
6. During home-based treatment, I pay attention to personal hygiene to reduce the risk of transmission to others (e.g., covering my mouth and nose when coughing).	363 (71.32%)	99 (19.45%)	33 (6.48%)	11 (2.16%)	3 (0.59%)

7. Before my sputum tests negative, I avoid sharing a bed with family members during home-based treatment.	348 (68.37%)	87 (17.09%)	53 (10.41%)	16 (3.14%)	5 (0.98%)
8. During home-based treatment, I use a covered sputum cup or spittoon with disinfectant at home.	316 (62.08%)	94 (18.47%)	60 (11.79%)	31 (6.09%)	8 (1.57%)
