# Supplementary material for: Knowledge, Attitudes, and Practices of Pulmonary Tuberculosis Patients Regarding Tuberculosis and Home-Based Treatment
Source: Healthcare (Basel). 2026 Aug 27;14(17):2732. doi: 10.3390/healthcare14172732 (PMC13565246; doi:10.3390/healthcare14172732)
Supplement: Supplementary file 1 [file healthcare-14-02732-s001.zip › healthcare-4413577 - Supplementary File S1- Final Questionnaire in English.pdf]

Questionnaire Number:

Dear Friend,

We are researchers from ** Hospital and sincerely invite you to participate in our research project. This study aims to understand **the knowledge, attitudes, and practices of patients with pulmonary tuberculosis regarding the disease and home-based treatment.** The findings will provide a basis for developing scientifically sound early intervention strategies, which may help more people in the future and improve patients' health conditions. Your participation in this study is entirely voluntary. If you agree to participate, please read the following information.

1. Please complete the questionnaire. There are no right or wrong answers; simply respond based on your actual situation. If you have any questions during the process, feel free to ask us. After completing the questionnaire, please submit it promptly.

2. This is a simple questionnaire survey and will not cause any harm to your physical or psychological health. However, it does involve some personal information such as your gender and age. We will strictly maintain confidentiality and will not disclose your information, so please feel free to answer truthfully.

3. As a participant, you may inquire about relevant information and the progress of this research at any time. If you decide to withdraw from the study, please inform us, and your data will not be included in the research results.

Finally, we sincerely thank you for taking the time to support our scientific research!

☐I have read and agree that the collected data will be used for scientific research.

Informed Consent Signature:

Date of Participation: Year Month Day

Part 1 Basic Information

**1. Your age (years): Height: cm Weight: kg (1 kg = 2 jin)**	
**2. Your gender:** a. Male b. Female	
**3. Your place of residence:**	a. Urban<br>b. Rural
**4. Ethnicity:**	a. Han<br>b. Ethnic minority
**5. Marital status:**	a. Single<br>b. Married<br>c. Divorced or separated<br>d. Widowed
**6. Your educational level:**	a. Primary school or below<br>b. Junior high school<br>c. Senior high school / Vocational school

	d. Associate degree / Bachelor’s degree
**7. Current employment status:**	a. Worker / Farmer<br>b. Self-employed<br>c. Government, enterprise, or public institution staff<br>d. Student<br>e. Other
**8. Your average monthly household income (RMB):**	a.<5000<br>b.5000-10000<br>c.10001-20000<br>d.>20000
**9. Living situation after discharge:**	a. Living alone<br>b. Living with family
**10. Was there a delay in tuberculosis diagnosis? (i.e., more than 4 weeks between onset of coughing and first hospital**	a. Yes<br>b. No

**visit for diagnosis indicates delayed diagnosis)**	
**11. Are you a newly diagnosed (initial treatment) patient?**	a. Yes<br>b. No

Part 2: Knowledge of Tuberculosis and Home-Based Treatment

**1. Tuberculosis is an infectious disease caused by bacteria.**	A．Yes	B．No	C．Not sure
**2. Which part of the body is most commonly affected by tuberculosis?**	A．Bones and joints	B．Lungs	C. Cardiovascular system
**3. What is the main route of transmission for pulmonary tuberculosis?**	A．Contact transmission	B．Respiratory transmission	C．Contaminated water or food
**4. Can pulmonary tuberculosis be cured?**	A．Yes	B．No	C．Not sure
**5. Can a person with smear-positive pulmonary tuberculosis transmit the disease to friends or family members?**	A．Yes	B．No	C．Not sure
**6. How long is the standard treatment period for regular pulmonary tuberculosis?**	A．3 months	B．6~8 months	C．18~24 months
**7. How long is the standard treatment period for drug-resistant tuberculosis?**	A．6~8 months	B．18~24 months	D．12-36 months

**8. Tuberculosis patients who are discharged from the hospital before completing their treatment course should continue the anti-tuberculosis treatment plan prescribed during hospitalization at home.**	A．Yes	B．No	C．Not sure
**9. During home-based treatment, patients do not need medical guidance from healthcare professionals.**	A．True	B．False	C．Not sure
**10. During home-based treatment, patients can adjust their medication dosage by themselves if symptoms improve or worsen.**	A．True	B．False	C．Not sure
**11. If a patient's home-based treatment is not properly managed, it may lead to treatment failure or the development of drug-resistant tuberculosis.**	A．True	B．False	C．Not sure
**12. Can an infectious tuberculosis patient share a bed with family members before their sputum tests turn**	A．Yes	B．No	C．Not sure

negative?			
13. **During home-based treatment, if an active tuberculosis patient wears a mask in shared household areas, maintaining a distance of more than 2 meters is unnecessary.**	A．Yes	B．No	C．Not sure
14. **Patients should take measures during home-based treatment to prevent and reduce the transmission of tuberculosis to close household contacts.**	A．Yes	B．No	C．Not sure
15. **Timely disinfection of the living environment and waste during home-based treatment can reduce the risk of transmission to family members and the community.**	A．Yes	B．No	C．Not sure

Part 3 Attitudes Toward Tuberculosis and Home-Based Treatment

**1. I feel ashamed because I have tuberculosis.**	A. strongly agree	B. agree	C. neutral	D. disagree	E . strongly disagree
**2. I believe my tuberculosis can be completely cured.**	A. strongly agree	B. agree	C. neutral	D. disagree	E . strongly disagree
**3. I feel confident in the safety of home-based treatment and am not worried about my condition worsening.**	A. strongly agree	B. agree	C. neutral	D. disagree	E . strongly disagree
**4. I believe that as long as I strictly follow the treatment plan prescribed by my doctor, home-based treatment can ensure timely recovery.**	A. strongly agree	B. agree	C. neutral	D. disagree	E . strongly disagree
**5. I believe that home-based treatment can help reduce the burden on healthcare resources to some extent.**	A. strongly agree	B. agree	C. neutral	D. disagree	E . strongly disagree
**6. I am willing to actively follow the doctor’s recommendations, take my medications on time, and attend regular follow-up visits.**	A. strongly agree	B. agree	C. neutral	D. disagree	E . strongly disagree

**7. Without close supervision by a doctor, I find it difficult to strictly follow the treatment plan during home-based treatment.**	A. strongly agree	B. agree	C. neutral	D. disagree	E . strongly disagree
**8. I believe that home-based treatment may lack proper infection control and pose a risk of transmitting the disease to my family.**	A. strongly agree	B. agree	C. neutral	D. disagree	E . strongly disagree
**9. If conditions allow, I would prefer to complete treatment in a hospital rather than at home.**	A. strongly agree	B. agree	C. neutral	D. disagree	E . strongly disagree

Part 4 Practices Regarding Tuberculosis and Home-Based Treatment

**1. During home-based treatment, I take my medication exactly as prescribed by my doctor.**	A．always able to do<br>B．usually able to do<br>C．not sure<br>D．sometimes unable to do<br>E．completely unable to do
**2. During home-based treatment, I never forget to take my medication.**	A．always able to do<br>B．usually able to do<br>C．not sure<br>D．sometimes unable to do<br>E．completely unable to do
**3. During home-based treatment, I do not stop taking medication just because my condition improves or worsens.**	A．always able to do

	B．usually able to do<br>C．not sure<br>D．sometimes unable to do<br>E．completely unable to do
**4. During home-based treatment, I will promptly contact the appropriate local institution if a treatment change is needed.**	A．always able to do<br>B．usually able to do<br>C．not sure<br>D．sometimes unable to do<br>E．completely unable to do
**5. During home-based treatment, I cooperate with relevant personnel to document my medication use in a timely manner.**	A．always able to do<br>B．usually able to do<br>C．not sure

	D．sometimes unable to do<br>E．completely unable to do
**6. During home-based treatment, I pay attention to personal hygiene to reduce the risk of transmission to others (e.g., covering my mouth and nose when coughing).**	A．always able to do<br>B．usually able to do<br>C．not sure<br>D．sometimes unable to do<br>E．completely unable to do
**7. Before my sputum tests negative, I avoid sharing a bed with family members during home-based treatment.**	A．always able to do<br>B．usually able to do<br>C．not sure<br>D．sometimes unable to do<br>E．completely unable to do

**8. During home-based treatment, I use a covered sputum cup or spittoon with disinfectant at home.**	A．always able to do<br>B．usually able to do<br>C．not sure<br>D．sometimes unable to do<br>E．completely unable to do
